# Non-invasive continuous real-time *in vivo* analysis of microbial hydrogen production shows adaptation to fermentable carbohydrates in mice

**DOI:** 10.1038/s41598-018-33619-0

**Published:** 2018-10-18

**Authors:** José M. S. Fernández-Calleja, Prokopis Konstanti, Hans J. M. Swarts, Lianne M. S. Bouwman, Vicenta Garcia-Campayo, Nils Billecke, Annemarie Oosting, Hauke Smidt, Jaap Keijer, Evert M. van Schothorst

**Affiliations:** 1 Human and Animal Physiology, Wageningen University, De Elst 1, Wageningen, 6708 WD The Netherlands; 2 Laboratory of Microbiology, Wageningen University, Stippeneng 4, Wageningen, 6708 WE The Netherlands; 3 Cargill R&D, Minneapolis, MN USA; 4 Cargill R&D Centre Europe, Havenstraat 84, Vilvoorde, 1600 Belgium; 5 Danone Nutricia Research, Uppsalalaan 12, Utrecht, 3584 CT The Netherlands

## Abstract

Real time *in vivo* methods are needed to better understand the interplay between diet and the gastrointestinal microbiota. Therefore, a rodent indirect calorimetry system was equipped with hydrogen (H_2_) and methane (CH_4_) sensors. H_2_ production was readily detected in C57BL/6J mice and followed a circadian rhythm. H_2_ production was increased within 12 hours after first exposure to a lowly-digestible starch diet (LDD) compared to a highly-digestible starch diet (HDD). Marked differences were observed in the faecal microbiota of animals fed the LDD and HDD diets. H_2_ was identified as a key variable explaining the variation in microbial communities, with specific taxa (including *Bacteroides* and *Parasutterella*) correlating with H_2_ production upon LDD-feeding. CH_4_ production was undetectable which was in line with absence of CH_4_ producers in the gut. We conclude that real-time *in vivo* monitoring of gases provides a non-invasive time-resolved system to explore the interplay between nutrition and gut microbes in a mouse model, and demonstrates potential for translation to other animal models and human studies.

## Introduction

Carbohydrates are a major dietary constituent of humans and rodents. Not all carbohydrates are metabolically equal. Most dietary carbohydrates, including several sugars and starches high in amylopectin content, are readily digested and thus absorbed early in the gastro-intestinal tract, making them quickly available to the organism^1^. Other carbohydrates, such as amylose-rich starches, are only available to the organism after fermentation by the intestinal microbiota^2^, which results in a more gradual release to the organism. Microbial fermentation results in a variety of metabolic products, including short-chain fatty acids (SCFA), which are thought to mediate the beneficial health effects of the intestinal microbial community^3^. Glucose and other monosaccharides, present as such in the diet or becoming available from highly-digestible carbohydrates, are readily taken up via transporters from the small intestinal lumen into the body. This occurs primarily in the jejunum, the proximal part of the small intestine^4^. Carbohydrates that are less readily digestible reach the caecum and colon, where most of the intestinal microbiota reside^5^. Specific microbial communities utilize these substrates, in the process generating metabolites that are absorbed by the body, or are excreted as gases or in the faeces. Major digestion products are SCFA, which are known to influence host physiology, acting as energy substrates and as signalling molecules^3^. Other digestion products are the microbial fermentation gases hydrogen (H_2_), methane (CH_4_), and hydrogen sulphide (H_2_S)^6^.

Since the studies of Gordon *et al*.^7^, it is increasingly realized that the small and large intestinal microbiota not only plays a major role in gastrointestinal health but also in the host’s metabolic health^8,9^. However, how the microbial community affects metabolic health and how this can be beneficially modulated by nutrition and specific nutrients is far less well established. While a variety of cross-sectional methods can be applied to analyse changes in intestinal microbiota in rodents at specific time points, longitudinal measurements in rodent and human studies mainly rely on sampling of the faeces, long after food-microbiota interactions have already taken place throughout the gastrointestinal tract. Continuous measurements of fermentation gas emissions are already in place for ruminants like cattle and sheep^10–12^, as they are known to fully rely on microbiota fermentation in rumen and hindgut to digest cellulose, being distinct from monogastric organisms including rodents and humans. Furthermore, recent studies showed strong correlations between dynamics of metabolite production and microbiota composition and activity in dairy cows^13,14^. Measurements of H_2_ and CH_4_ as indicators of human gut microbial activity *in vivo* have been used before^15–19^, but these are in fact single-time-point gas measurements that lack the information that continuous analysis can provide.

Therefore, our study objective was to apply a simple non-invasive method to monitor the effect of diet on intestinal microbiota in real time using a human-relevant model, which we envisioned as a powerful tool to better understand the direct impact of nutrition on the microbiota and by extension of diet-microbiota interactions on human health.

C57BL/6J mice are the most widely used model in medical and nutritional health research and have shown their validity in dissecting microbe-host interactions and causality testing. However, analysis of fermentation gases in mice and other rodent models is a largely unexplored area. As is the case in humans, single-time-point measurements of H_2_ (refs^20–23^) and CH_4_ (refs^24–26^) have been reported for mice and rats. This is critical, because only continuous measurements allow to faithfully study the time-resolved kinetics of digestion and metabolism of nutrients reaching the gut microbiota.

Indirect calorimetry makes use of the measurement of oxygen (O_2_) and carbon dioxide (CO_2_), as well as food and water intake and locomotor activity, to analyse energy metabolism. We have equipped a commercially available indirect calorimetry system with sensors for H_2_ and CH_4_, allowing continuous measurements of release of these gases non-invasively in real time. We applied this extended system to explore the adaptation of gut microbiota to highly- and lowly-digestible carbohydrates. To the best of our knowledge, this is the first time that food-microbiota interactions have been studied continuously, non-invasively and in real time in a murine model.

## Results

### *In vitro* reflects *in vivo* diet digestibility

**Figure 1:**
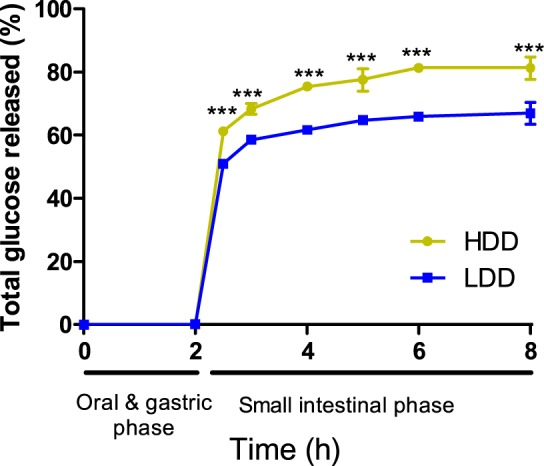
*In vitro* digestibility of starches in experimental diets. Triplicate samples of the lowly- and highly-digestible starch diets (LDD and HDD, respectively) were digested *in vitro*, and free glucose concentrations were determined at indicated time points. Statistical comparisons were made with two-way ANOVA with Bonferroni’s post hoc test; ***P ≤ 0.001. Values are plotted as mean ± s.d.

To confirm the difference in digestibility of the two starches incorporated into our experimental diets (Table 1), an *in vitro* model that mimics food digestion for the oral, gastric and small intestinal phases was used. The lowly-digestible starch diet (LDD) showed a slower and 14% less complete carbohydrate digestion than the highly-digestible starch diet (HDD; Fig. 1). In addition, we quantified food intake and faecal energy content in female and male mice habituated to the experimental diets (Table 2). Daily faecal mass was increased in all mice fed LDD, whereas faecal energy density was increased in LDD females only. LDD mice lost on average twice as much energy in faeces compared to HDD mice. With similar food and energy intake, the diet digestibility was 6% lower in LDD *vs* HDD fed mice (Table 2). Taken together, both *in vitro* and *in vivo* analyses showed a reduced digestibility of the LDD *vs* HDD.

**Table 1:** Diet composition.

Component	Diet	
	HDD	LDD
Casein	212.2	212.2
L-Cysteine	3.0	3.0
Amylose mix (AmyloGel 03003)	0.0	568.6
Amylopectin (C*Gel 04201)	568.6	0.0
Coconut oil	21.4	21.4
Sunflower oil	83.1	83.1
Flaxseed oil	14.2	14.2
Cholesterol	0.03	0.03
Cellulose	50.0	50.0
Mineral mix (AIN-93G-MX)	35.0	35.0
Vitamin mix (AIN-93-VX)	10.0	10.0
Choline bitartrate	2.5	2.5
Total (g)	1000.0	1000.0
Gross energy density (kJ g^−1^)^a^	18.9	19.5
Calculated energy density (kJ g^−1^)^b^	17.9	17.9
Protein (en%)^b^	20	20
Carbohydrate (en%)^b^	55	55
Fat (en%)^b^	25	25

Values are g kg^−1^ of diet unless otherwise specified. ^a^Measured by bomb calorimetry, ^b^calculated based on Atwater’s nutritional values. HDD, highly-digestible starch diet; LDD, lowly-digestible starch diet.

**Table 2:** Dietary *in vivo* digestibility of the experimental diets.

	Females			Males		
	HDD	LDD	*P* value	HDD	LDD	*P* value
Food intake (g)	2.53 ± 0.05	2.71 ± 0.24	0.1942	2.82 ± 0.21	2.86 ± 0.37	0.8489
Gross energy intake (kJ)	48.01 ± 0.92	53.08 ± 4.76	0.0816	53.36 ± 4.01	55.88 ± 7.18	0.5634
Faeces weight (g)	0.20 ± 0.01	0.41 ± 0.06	**0**.**0006**	0.24 ± 0.02	0.45 ± 0.05	**0**.**0002**
Faeces gross energy (kJ g^−1^)	15.48 ± 0.26	16.18 ± 0.24	**0**.**0072**	15.94 ± 0.27	16.01 ± 0.23	0.7227
Faeces energy loss (kJ)	3.10 ± 0.12	6.68 ± 1.08	**0**.**0006**	3.74 ± 0.22	7.26 ± 0.78	**0**.**0001**
Digestible energy intake (kJ)	44.91 ± 0.91	46.40 ± 3.70	0.4645	49.63 ± 3.80	48.62 ± 6.44	0.7967
Diet digestibility (%)	93.6 ± 0.2	87.5 ± 0.9	**<0**.**0001**	93.0 ± 0.2	87.0 ± 0.6	**<0**.**0001**

Energy balance calculated over the third week of exposure to the diets in females and males (n = 4 per sex and diet) and expressed per day. Statistical comparisons by Student’s *t*-test within sex. Data reported as mean ± s.d.

### Measuring H_2_ production in real time

**Figure 2:**
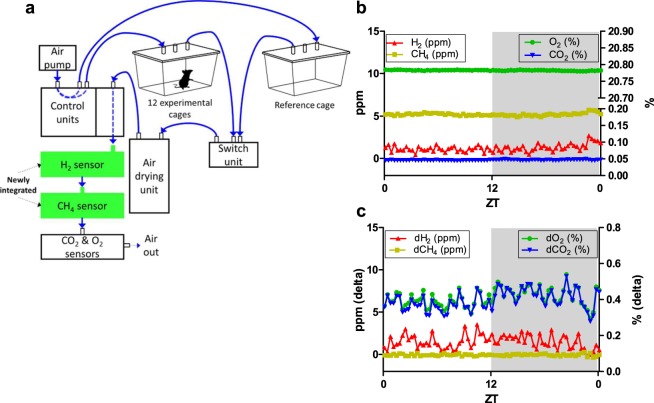
Real-time measurements of hydrogen (H_2_) and methane (CH_4_) production in mice within indirect calorimetry system. (**a**) Illustration of the indirect calorimetry system extended for H_2_ and CH_4_ measurements. Direction of air flow in the tubing is shown in blue, new gas sensors are shown in green. For clarity, tube lengths are not to scale (all equal) and food and drink containers with sensors are not shown, nor are the infrared beam bars for activity measurements. (**b**) Ambient concentrations of H_2_ and CH_4_ (left y-axis, ppm) and O_2_ and CO_2_ (right y-axis, %) were recorded in an empty (reference) cage at 20 min intervals for 24 h. (**c**) Gas concentrations in a cage occupied by a chow-fed female adult mouse were measured and compared to the corresponding concentrations in the reference cage and expressed as delta values. White and grey areas in panels b and c represent the inactive light and active dark phase for the animal, respectively. ZT, Zeitgeber time.

Reduced digestibility likely also affects colonic fermentation, for which H_2_ has been used as a marker in mice^20^. However, measurement of its continuous production in response to the diet has not yet been possible. We therefore adapted and extended an indirect calorimetry system to allow H_2_ and CH_4_ production to be studied in real time, by introducing the respective sensors in series with the O_2_ and CO_2_ sensors already present in the system (Fig. 2a). To determine if the small quantities of H_2_ originating from microbial carbohydrate fermentation in mice could be detected by our system, we measured gas concentrations in cages with and without chow-fed mice over 24 h. Stable signals for all gases were seen in the absence of mice (Fig. 2b), and the concentrations were clearly decreased for O_2_ and increased for CO_2_ in mouse-occupied cages, as expected (Fig. 2c). H_2_ increased (Fig. 2c), while CH_4_ concentrations were not altered by the presence of a chow-fed mouse in the cage. The adapted indirect calorimetry system was therefore suitable for simultaneous respirometry and H_2_ production measurements in real time, however under the conditions tested, CH_4_ production appeared to be absent based on measured ambient levels well above the lower detection limit of the CH_4_ sensor (Fig. 2b).

### H_2_ production indicates extent of carbohydrate digestibility

Since the contrasting digestibility of the experimental diets was expected to result in sustained differences in H_2_ production as a consequence of fermentation in the large intestine, we fed female and male mice, as a proof-of-concept, either the HDD or the LDD for three weeks and measured H_2_, CH_4_, O_2_, and CO_2_ levels continuously during several days (Study 1). Calculation of energy expenditure, based on 24 h O_2_ consumption and CO_2_ production, revealed no differences between dietary groups (females 1.59 ± 0.08 *vs* 1.63 ± 0.08 kJ h^−1^ in HDD and LDD respectively, *P* = 0.2094; males 1.80 ± 0.13 *vs* 1.76 ± 0.13 kJ h^−1^ in HDD and LDD respectively, *P* = 0.5470). However, 24 h mean respiratory exchange ratio (RER) was lower in LDD- *vs* HDD-fed male mice (0.85 ± 0.03 *vs* 0.88 ± 0.03 respectively, *P* = 0.0097), indicating higher fat oxidation and lower carbohydrate oxidation in LDD mice. Overall, these observations agree with indirect calorimetry data reported for mice fed diets containing carbohydrates similar to the carbohydrates used here^27^.

**Figure 3:**
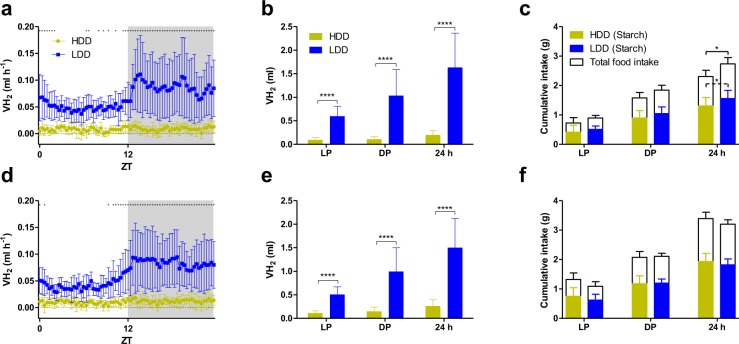
H_2_ production in mice reflects starch digestibility. Female (**a**) and male (**d**) mice were fed either HDD or LDD for three weeks and volume of H_2_ produced (VH_2_) was recorded for 24 h in the adapted indirect calorimetry system. Cumulative H_2_ production in females (**b**) and males (**e**) quantified during the 12 h light phase (LP), 12 h dark phase (DP) or the complete 24 h photoperiod. Cumulative starch and total food intake in females (**c**) and males (**f**) over the measuring period calculated from food intake records. White and grey areas represent the light and the dark phase, respectively. Time course data was analysed by repeated measures two-way ANOVA with Bonferroni’s test for multiple comparisons and time points where *P* < 0.05 are indicated with black asterisks (panels a and d). Other statistical comparisons made by Student’s *t*-test or Mann-Whitney *U* test; *P ≤ 0.05, ****P < 0.0001 (*n* = 11 LDD females, *n* = 12 remaining groups). Data shown as mean ± s.d. ZT, Zeitgeber time.

Both LDD-fed females (Fig. 3a,b) and males (Fig. 3d,e) constantly produced more H_2_ than HDD-fed mice. A distinct pattern of H_2_ production became apparent in LDD-fed mice, with H_2_ levels being higher in the active dark phase and lower, but still clearly present, in the inactive light phase (Fig. 3a,b,d,e). This was fully consistent with the circadian food and starch intake (Fig. 3c,f). Importantly, the difference in H_2_ production between HDD- and LDD-fed mice was explained by the type of starch rather than the amount of starches ingested, as cumulative starch consumption was similar between the groups (Fig. 3c,f). Together, this data provides proof-of-concept for measuring H_2_ production in real time as an indicator of carbohydrate digestibility.

### H_2_ evolution reflects adaptation to dietary carbohydrates

As we could show that H_2_ production can be sensitively and continuously measured, we next questioned whether it would be possible to measure adaptation to the diet *in vivo* in real time.

**Figure 4:**
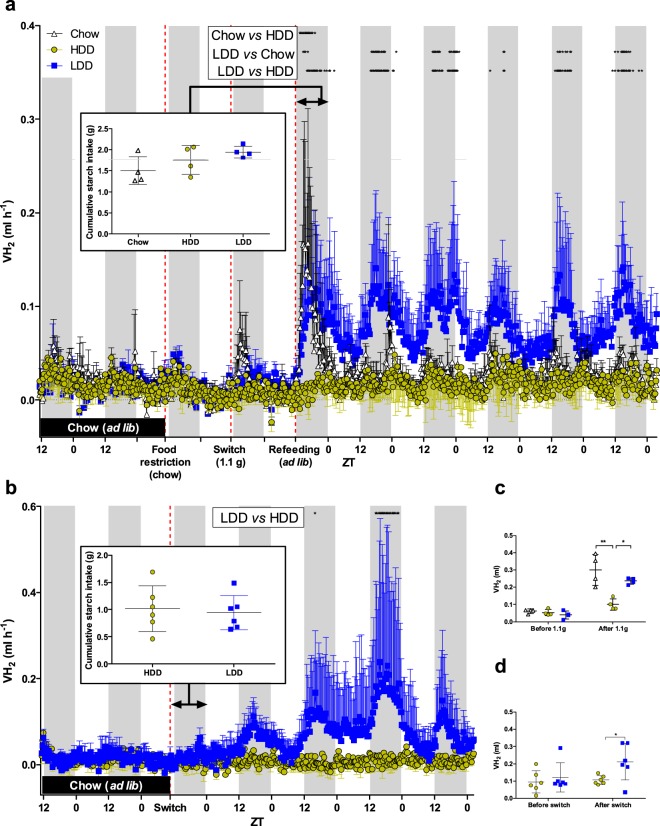
H_2_ evolution upon first exposure to starches of different digestibility. (**a**) Standard chow-fed mice within indirect calorimetry were food-restricted leading to fasting (dotted line), which was followed by feeding 1.1 g of chow (black), HDD (yellow), or LDD (blue; *n* = 4 per group) prior to the dark phase as a single meal test (2^nd^ dotted line). As a result, they were fasted the next day, and received prior to dark phase *ad libitum* access to the same diet (3^rd^ dotted line) for an additional 5.5 days. Inset: First 12 h cumulative starch-intake of *ad libitum* feeding with experimental diets. (**b**) Chow-fed mice (*n* = 6 per group) were switched to LDD or HDD without prior food restriction and measurements continued for another 4.5 days. Inset: First 12 h cumulative starch-intake after diet switch. (**c**) Cumulative H_2_ production over 12 h before (while food-restricted on chow) and after feeding 1.1 g of chow, HDD, or LDD (*n* = 4 per group). (**d**) Cumulative H_2_ production over 12 h before and after switching directly from chow to HDD or LDD (*n* = 6 per group). All mice received no other diet than chow during their whole lifetime prior to these experiments and the dietary switch (black bar), but colour usage reflects subgroups after first exposure to new diets. White and grey areas represent light and dark phases, respectively. Time course data was analysed by repeated measures two-way ANOVA with Bonferroni’s test for multiple comparisons (chow *vs* HDD, LDD *vs* chow, and LDD *vs* HDD), and time points where *P* < 0.05 are indicated with black stars (panels a,b). Cumulative data was statistically compared using unpaired two-tailed Student’s *t*-test (between HDD and LDD) and one-way ANOVA with Bonferroni’s multiple comparisons *post hoc* test (between chow, HDD, and LDD); *P ≤ 0.05, **P ≤ 0.01. Data is presented as mean ± s.d. For clarity, either upper or lower error bars are shown. ZT, Zeitgeber time.

For this, we provided HDD or LDD to mice that had no previous exposure to these diets and we followed H_2_ production continuously. We introduced the new diets in one of two conditions; the first condition was as a single meal challenge given to fasted mice, followed by *ad libitum* access to the diet the next day, as a second fasting-refeeding challenge (Study 2, Fig. 4a). The second condition was by replacing the standard chow diet directly with HDD or LDD *ad libitum* (Study 3, Fig. 4b). H_2_ production was significantly increased in LDD- compared to HDD-fed mice as early as 4 h after fasted mice gained *ad libitum* access to the experimental diet (Fig. 4a). The direct switch from chow to HDD or LDD without fasting had similar results, with LDD-fed mice producing significantly more H_2_ after 53 h of access to the LDD compared to mice receiving HDD (2-way ANOVA, Fig. 4b). In both conditions, *i*.*e*. fasted or directly switched to HDD or LDD, cumulative H_2_ production became significantly higher already within 12 h upon access to LDD *vs* HDD (Fig. 4c,d), and H_2_ production patterns in LDD-fed mice closely followed the patterns of LDD intake (Additional File 1: Fig. S1). Interestingly, mice that continued on the chow diet after a period of food restriction exhibited a spike in H_2_ production (Fig. 4a), while consuming similar amounts of starches compared to the HDD and the LDD groups. H_2_ production in HDD-fed mice remained lower compared to mice on LDD or chow, as expected. Importantly, LDD-induced H_2_ production increased gradually before reaching its maximal levels (up to 0.89 ml h^−1^), revealing the process of adaptation to the lowly-digestible starch. LDD- *vs* HDD-fed mice thus showed a differential adaptation, likely in their microbiota, based on increased H_2_ production.

### Alterations in intestinal microbiota by dietary carbohydrates

**Figure 5:**
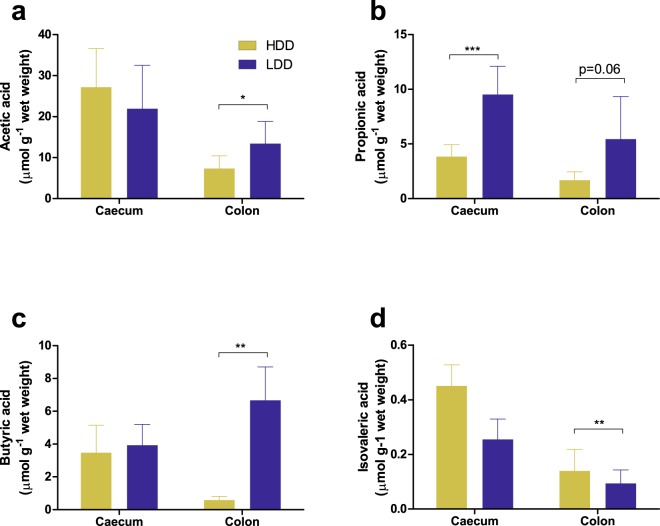
Short-chain fatty acid (SCFA) concentrations in intestinal digesta of mice fed starches of different digestibility. (**a**) Acetic acid, (**b**) propionic acid, (**c**) butyric acid and (**d**) isovaleric acid concentrations in mouse caecum (*n* = 6 per group) and colon (*n* = 5 HDD, *n* = 7 LDD) contents obtained after three weeks of feeding HDD (yellow bars) or LDD (blue bars). Statistical comparisons were made using unpaired two-tailed Student’s *t*-test; *P ≤ 0.05, **P ≤ 0.01, ***P ≤ 0.001, ****P < 0.0001. Data shown as mean ± s.d.

Since the production of H_2_ fully depends on intestinal microbial communities and their metabolism, we further investigated the changes in the microbiota induced by the LDD to validate our observations. As an additional parameter of fermentation, we first assessed SCFA levels in intestinal digesta after 3 weeks of exposure to the HDD or the LDD (Study 1). Total caecal SCFA levels were similar between LDD- and HDD-fed mice (35.6 ± 13.9 *vs* 34.9 ± 11.9 μmol g^−1^, respectively), including valeric and isobutyric levels (data not shown), whereas total SCFA in colon were higher in LDD- compared to HDD-fed mice (25.6 ± 9.6 *vs* 9.6 ± 4.1 μmol g^−1^, *P* = 0.0059). Acetic acid (Fig. 5a) and propionic acid (Fig. 5b) were the two most abundant SCFA, and both were significantly elevated in LDD-fed mice in colon and caecum contents, respectively. Butyric acid was the most differentially produced SCFA, enriched by 13.8-fold in LDD colon content (Fig. 5c). Finally, isovaleric acid, a product of microbial protein fermentation, was the least abundant of the measured SCFA in all groups and was significantly lower in caecum of LDD- *vs* HDD-fed mice (Fig. 5d).

**Figure 6:**
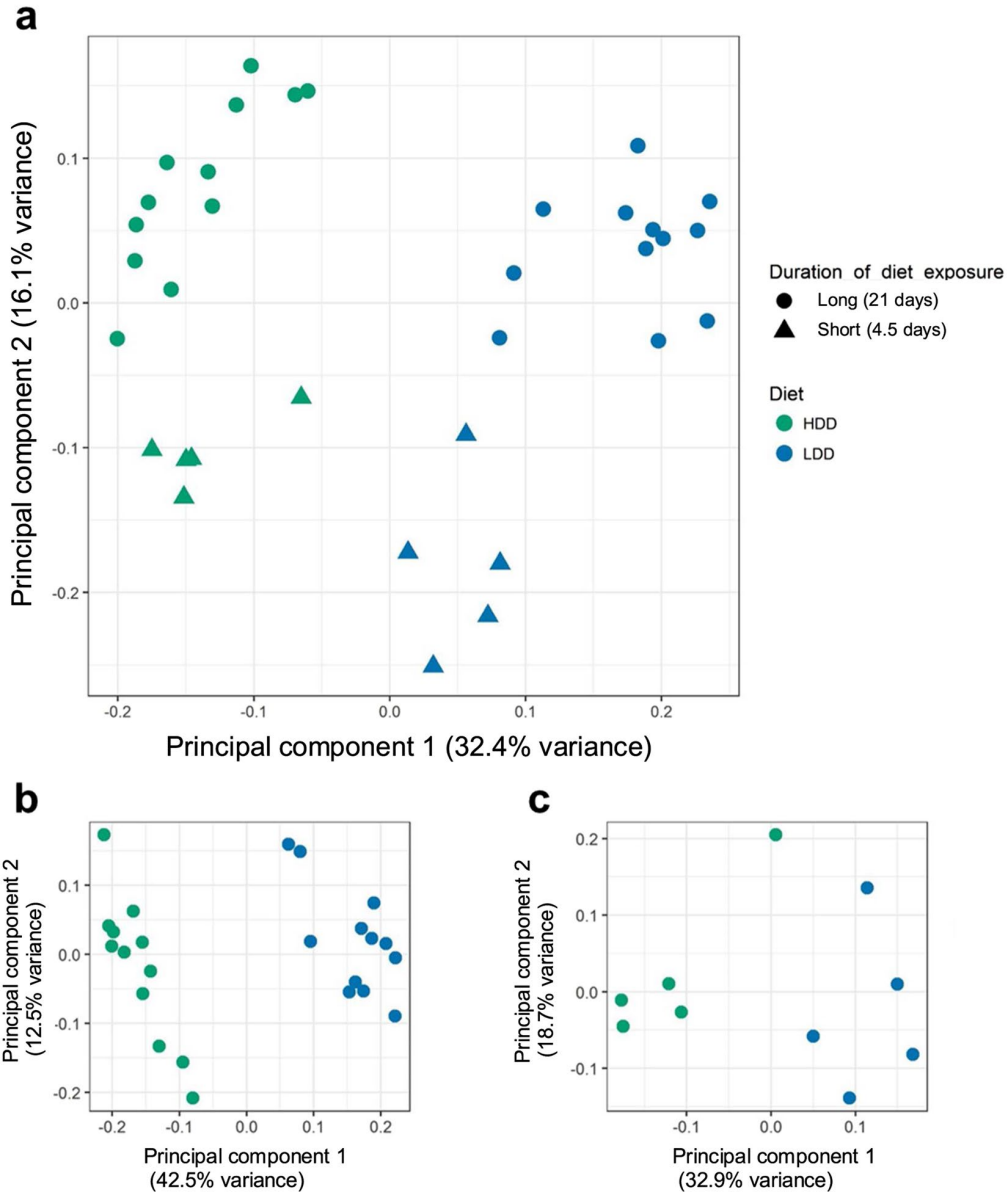
Starch digestibility primarily determines faecal microbiota composition. Principal coordinates analysis (PCoA) plot illustrating the unweighted UniFrac distances of the intestinal microbiota of mice after long- and short-term exposure to HDD and LDD (**a**, Studies 1 and 3 combined), and only long-term (**b**, Study 1) and short-term (**c**, Study 3) exposures. Each data point represents a sample of faecal pellets of one individual mouse (*n* = 12 long-term exposure per diet, *n* = 5 short-term exposure per diet).

We next compared the overall changes in faecal microbiota communities induced by HDD or LDD after exposure to the diets for 3 weeks (Study 1) and 4.5 d (Study 3). Principal coordinates analysis (PCoA) using the UniFrac unweighted distance matrix revealed a clear separation between the two dietary groups (Fig. 6a). These observations were supported by Adonis analysis, using either weighted or unweighted UniFrac distances, as diet explained a large part of the variation in microbiota composition (20% and 29%, respectively, *P* < 0.001, Table 3). H_2_ volume was the second most important variable, followed by duration of intervention and age, and body weight, with minor but significant effects (Table 3). Of note, duration of intervention and age of mice are dependent variables due to study design. In order to control for the effects of duration of dietary exposure, we also analysed Studies 1 and 3 separately. After 3 weeks of intervention, diet and H_2_ production were the only significant variables, with H_2_ explaining up to 34% of the variation (Fig. 6b and Table 3). However, only diet contributed significantly to the variation after 4.5 d of intervention in adult mice (Fig. 6c and Table 3). Additionally, α-diversity appeared to decrease with duration of intervention irrespective of the dietary intervention, with no consistent effects of the diet (Additional File 2: Fig. S2). This is in line with the differences in age of these mice, namely the young mice showing lower α-diversity than the older mice.

**Table 3:** Faecal microbiota composition of mice fed HDD or LDD and other host and environmental variables.

	Weighted UniFrac		Unweighted UniFrac	
	R^2^	*P* value	R^2^	*P* value
**Studies 1 and 3 combined (long- and short-term exposures)**				
Diet	0.198	**0.001**	0.287	**0.001**
Cumulative H_2_ production	0.098	**0.023**	0.173	**0.001**
Duration of intervention*	0.094	**0.015**	0.141	**0.001**
Age*	0.094	**0.02**	0.141	**0.002**
Sex	0.057	0.12	0.05	**0.018**
Body weight	0.08	**0.042**	0.1	**0.002**
Food intake	0.06	0.08	0.043	0.142
Starch intake	0.068	0.079	0.043	0.135
**Study 1 (long-term exposure**, **post-weaning**, **n = 12)**				
Diet	0.26	**0.003**	0.4	**0.001**
Cumulative H_2_ production	0.198	**0.005**	0.344	**0.001**
Sex	0.062	0.195	0.03	0.658
Body weight	0.046	0.289	0.02	0.87
Food intake	0.099	0.062	0.049	0.3
Starch intake	0.1	0.077	0.05	0.279
**Study 3 (short-term exposure**, **adult**, **n = 5)**				
Diet	0.24	0.056	0.293	**0.004**
Cumulative H_2_ production	0.14	0.217	0.108	0.5
Body weight	0.08	0.594	0.137	0.228
Food intake	0.06	0.748	0.192	**0.044**
Starch intake	0.06	0.761	0.192	0.05

Results are obtained using Adonis Permutational Multivariate Analysis of Variance. *Duration of intervention (short- and long-term) and age of animals (young *vs* adult) are not independent variables.

**Figure 7:**
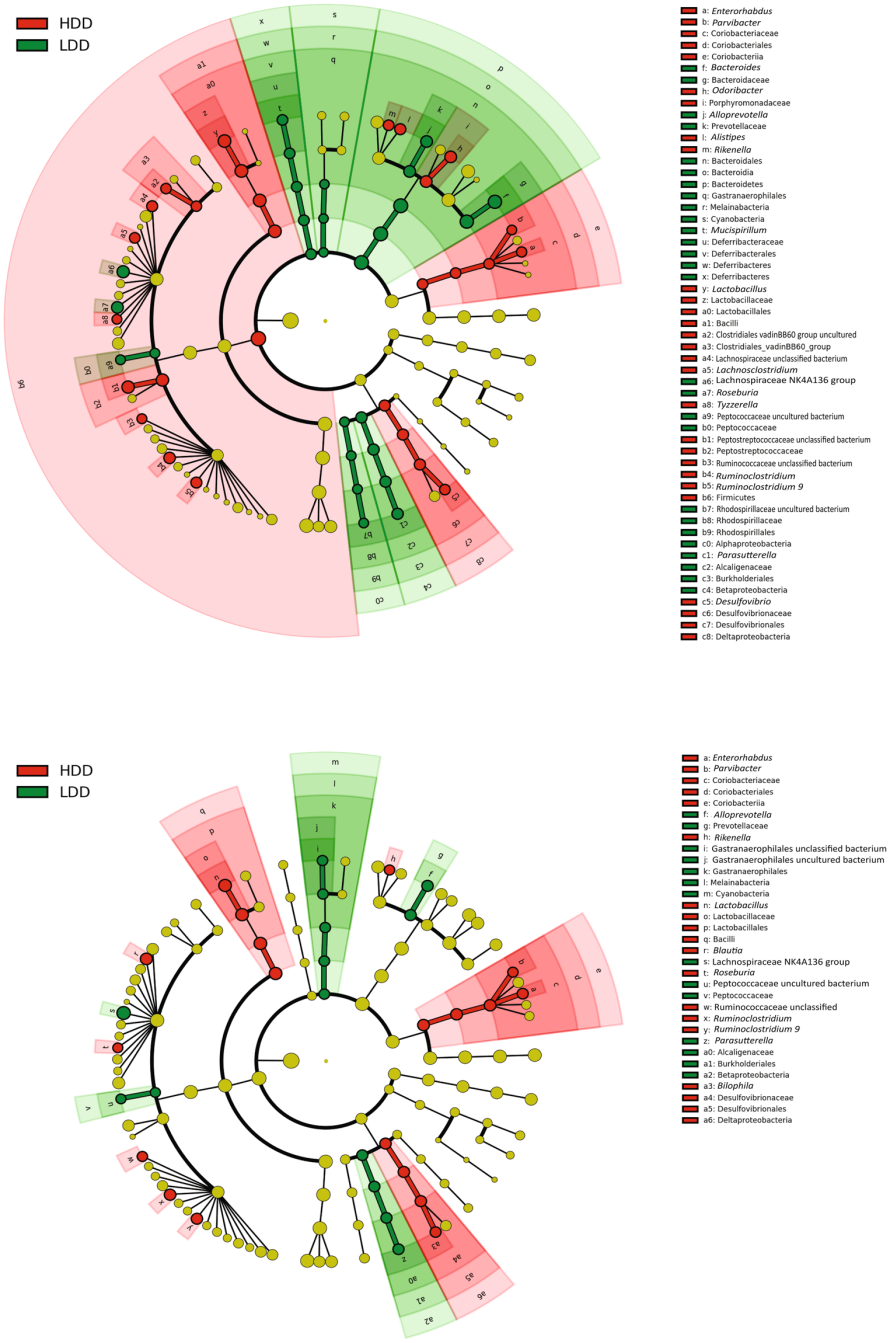
Exposure to starches of different digestibility induces distinct microbial taxa. (**a**) Cladogram representing bacteria genera that were significantly enriched by LDD or HDD after 3 weeks of exposure to the diets (*n* = 12 per diet, Study 1). (**b**) Bacterial genera that were significantly increased by LDD or HDD after 4.5 d of exposure to the diets (*n* = 5 per diet, Study 3). Comparisons were done using the linear discriminant analysis effect size (LEfSe) method. LDA scores are shown in Additional file 3: Fig. 3e,f. Nomenclature of microbial genus level taxa is based on highest achievable taxonomic resolution at phylum, class, order, family or genus level.

**Figure 8:**
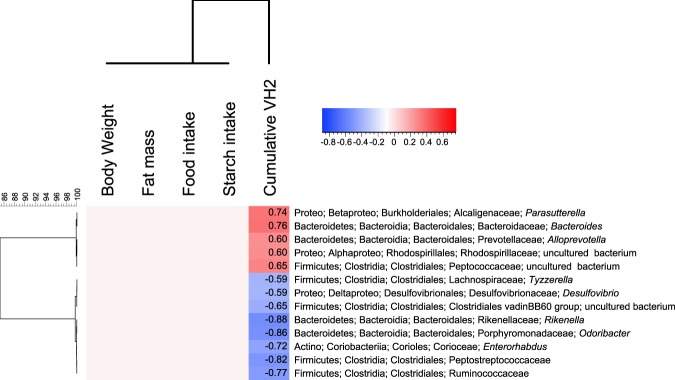
Specific bacterial genera correlate only with *in vivo* H_2_ production. Spearman’s rank correlation coefficients of faecal microbiota, H_2_ production, food and starch intake, body weight, and fat mass of mice exposed to HDD or LDD for 3 weeks after weaning (*n* = 12 per diet, Study 1). Non-red and non-blue cells all have a Spearman’s correlation value of 0 with FDR *P* value > 0.13. Nomenclature of microbial genus level taxa is based on highest achievable taxonomic resolution at phylum, class, order, family or genus level.

We then aimed to identify which microbial taxa were significantly associated with the observed differences in β-diversity. The microbiota of mice fed LDD *vs* HDD for 3 weeks was enriched in *Bacteroides*, *Parasutterella*, *Roseburia*, and *Alloprevotella*, along with two other families (Fig. 7a and Additional File 3: Fig. S3a). In comparison, *Lactobacillus*, *Rikenella*, *Odoribacter*, *Enterorhabdus*, and *Desulfovibrio* among others appeared enriched in HDD- *vs* LDD-fed mice (Fig. 7a and Additional File Fig. S3b). Similar differences were seen after 4.5 d of exposure (Fig. 7b and Additional file 3: Fig. S3c,d). While fewer taxa were affected by the short-term dietary intervention, changes in genus level were consistent for both groups (Additional file 4: Fig. S4). Moreover, H_2_ production was the only (environmental) variable that was significantly correlated with specific bacteria taxa after three weeks of intervention, with five genera correlating positively with H_2_ production and eight genera showing a negative correlation (Fig. 8 and Additional file 5: Fig. S5). Eleven of these 13 genera were also significantly influenced by diet (Fig. 7a and Additional file 3: Fig. 3a,b). Finally, Archaea (some of which are CH_4_ producers) could not be detected in any of the samples despite the use of primers targeting both bacterial and archaeal 16S rRNA genes equally well. This agrees with the absence of CH_4_ detection in these mice and under these nutritional challenges.

## Discussion

The goal of this study was to measure real-time interactions between diet, gut microbes, and the host. We implemented H_2_ and CH_4_ detection in an indirect calorimetry system to track fermentation continuously in mice. H_2_ production revealed a time frame for microbiota adaptation to starch of low digestibility, which corresponded with shifts in microbial community composition induced by diet. Thus, measuring H_2_ production allowed us to non-invasively study effects of the diet on the intestinal microbiota in real-time.

The difference in starch digestibility as part of the experimental diets was confirmed both by *in vitro* and *in vivo* measurements, but did not significantly alter total intake of digestible energy (gross energy minus faecal energy losses) between dietary groups within a sex. The lower digestibility of the starch in the LDD thus suggests that partially undigested starch reached the large intestine which was subsequently partially fermented by the intestinal microbiota providing energy substrates, *e*.*g*. SCFA, to the host. Energy of undigested starch can be lost after fermentation in the form of products not utilizable by the host, such as H_2_. However, previous studies considered energy loss in the form of H_2_ and CH_4_ negligible, representing less than 0.2% of total energy expenditure in humans consuming non-starch polysacharides^28^. Studies in rats fed various types of resistant starch also indicated that energy loss through fermentation is minimal, although the actual H_2_ output was not measured directly^29^. Here, our data show that H_2_ is produced constantly on a lowly-digestible starch diet. Although the volume of H_2_ produced by the mice in our study may be little in terms of energy loss, it is plausible that carbohydrates that give a higher level of fermentation could further increase the H_2_ output, which might represent a significant factor to take into account over a lifetime.

H_2_ production was detected in mice under all conditions tested, with the amounts produced clearly being influenced by the form of carbohydrate consumed. Mice fed moderately fermentable carbohydrates have been shown to produce H_2_ (ref.^20^). Even in conditions where little fermentation is expected, such as feeding corn starch-based chow^30^ or pure sucrose^31^, H_2_ production has been seen in rats. In line with our data on three diets with a different carbohydrate profile, this illustrates that H_2_ production can directly reflect subtle changes in carbohydrate fermentation. Interestingly, H_2_ production was clearly associated with the food intake pattern. This is in contrast with data reported in humans, where H_2_ and CH_4_ peaked at rather unpredictable times after food intake despite the proper control of the meal schedule^28^. This might be due to e.g. differences in dietary meal composition, time resolution of sampling, intestinal transit time, or other differences in intestinal physiology between humans and mice. More recently, using gut capsule technology, a similar H_2_ pattern as in our mice was also observed in a human pilot trial based on dietary fibre differences^32^.

We demonstrated that real-time monitoring of H_2_ production can be used to investigate transient effects of diet in time and explore the process of adaptation rather than the end stage only. So far, studies mainly investigated, by measuring H_2_ and other fermentation parameters at selected time points, longer timeframes ranging from 1 day to several weeks^25,33–35^. In our study, significant differences in H_2_ production appeared within 12 h upon access to LDD. This timeframe was clearly influenced by fasting and whether the diet was provided *ad libitum* or in a restricted amount. We speculate these early increases in H_2_ output to reflect immediate effects of diet on microbial metabolism preceding changes in community structure. Another observation was that mice fed chow produced H_2_, although at low levels. Real-time monitoring newly revealed that a period of food restriction followed by refeeding led to a marked and acute increase in H_2_ production once chow became available again. A likely explanation is excessive eating after food deprivation, causing a larger amount of not fully digested chyme to enter the large intestine and thus increasing substrate availability to the microbiota. In addition, a 24 h fasting period alone has been shown to produce shifts in microbial diversity^36^ and microbiota configuration^37^. Such changes could in turn alter fermentation stoichiometry and microbial function in response to the diet and ultimately lead to a higher H_2_ output. Our analysis indicates (short-term) effects of fasting and refeeding on microbial activity, which should be careful taken into account in nutritional studies focussing on changes in microbiota composition and function.

As could be expected, the driver of the experimental differences, the dietary starch digestibility, was the most important factor explaining the variation in microbiota, showing colinearity with our measured *in vivo* H_2_ production. Although current knowledge of the dynamics of H_2_ within the gastrointestinal tract is limited, it is well documented that H_2_ is exclusively produced during fermentation by hydrogenogens^6^. Among the major hydrogenogenic bacteria are Bacteroidetes and clostridial members of Firmicutes^38^. In line with this, we observed that LDD, a source of carbohydrates for caecum and colon, stimulated the fermentative Bacteroidetes bacteria, more specifically the genus *Bacteroides*. This is consistent with the dose-dependent increase in caecal Bacteroidetes density in response to amylose^39^ and similar findings for amylose on a high-fat background^40^. Here we extend these findings and show, for the first time *in vivo*, a positive correlation between *Bacteroides* and H_2_ production in mice.

Interestingly, after the short-term exposure to LDD in adult mice, Bacteroidetes were not significantly increased compared to the HDD group. This might be associated with the shorter duration of the treatment and possibly with more firmly established microbial communities in adulthood. However, most genera induced by diet in adult mice after 4.5 days correspond to those induced in mice after weaning, which were exposed for 3 weeks.

Another consistent shift in microbial community composition was the promotion of Deltaproteobacteria, particularly *Desulfovibrio* and *Bilophila*, in HDD-fed mice. Deltaproteobacteria are the major representatives of colonic sulphate reducing bacteria (SRB)^41^ including *Desulfovibrio*. SRB along with methanogens and acetogenic bacteria are the only gut microbes able to use H_2_ as an electron donor to produce H_2_S and acetate. Although not a SRB itself, taurine-respiring *Bilophila* species can also produce H_2_S. Additionally, there is evidence of CH_4_ production in rats^26^ and mice, with the presence of methanogens in humanized microbiota mouse models^42^ and by high fat dietary feeding^43^. The fact that we neither detected CH_4_ nor Archaea suggests that H_2_ was preferentially used to produce H_2_S in mice fed readily digestible starch. H_2_S is a potentially toxic product of bacterial metabolism^44–46^ and it has been implicated in human health and disease^19^ and, more recently, thermogenesis^47^. Moreover, H_2_S has been reported to inhibit the production of SCFA and specifically to impair butyrate oxidation, depriving colonic cells from their main energy source^45,48^. In line, we report a dramatic difference in colonic butyrate in HDD-fed mice. Apart from Deltaproteobacteria, we observed increased abundances of *Odoribacter*, a known H_2_S producer^49^ and *Rikenella*, a desulphatase-secreting bacterium^50^, under HDD-feeding. Members of the genus *Rikenella* are able to cleave sulphate from mucin glycans, making them available for microbial degradation^51^ and potentially acting as a donor of sulphate to H_2_S producers. Based on these facts we speculate that the lack of fermentable carbohydrates favoured the presence of hydrogenotrophs associated with the production of H_2_S, which could have led to the decreased H_2_ output and colonic SCFA levels that was observed in mice fed highly-digestible starch.

The major taxon increased in HDD-fed mice in our study belonged to the genus *Lactobacillus*. In contrast, diets supplemented with resistant starch tended to enrich the *Lactobacillus* population in mouse caecum, but much less at high doses of resistant starch^39^. Incidentally, hydrogenase genes, which encode enzymes for the reversible oxidation of H_2_, were recently shown to be completely absent in Bacilli and bifidobacteria^38^. Considering the lack of a correlation between H_2_ production and *Lactobacillus* in our study, new questions emerge about the ability of *Lactobacillus* to thrive in H_2_-poor environments.

The increase in isovaleric acid, a product of branched-chain amino acid catabolism^52^, in HDD-fed mice, suggests a shift of microbiota towards protein fermentation. Bacteria from the genera *Enterorhabdus*^53^ and *Parvibacter*^54^, both significantly induced by HDD-feeding, have the ability to ferment amino acids. Additionally *Olsenella*, only present in two samples in the HDD group, is documented to grow on tyrosine and produce *p*-cresol^55^, supporting our hypothesis for a shift to protein fermentation. This might have important implications for the host, since products of protein fermentation such as phenols, ammonia, certain amines, and H_2_S, are considered to play important roles in the initiation or progression of bowel diseases, inflammation, DNA damage, and cancer^56^.

Altogether, our results emphasize H_2_ as a key factor within the intestinal microenvironment and the usefulness of knowing its production dynamics to understand the interplay between host, diet, and the intestinal microbiota. At the same time, we are aware that our approach to study such interactions may have conceivable limitations. It has been argued that changes in gas evolution (and other indirect markers of fermentation) cannot accurately indicate changes in fermentation^57^, and even “real-time”, carefully controlled measurements have failed to show quantitative changes in H_2_ and CH_4_ production proportionally linked to consumption of fermentable carbohydrates^15,28^. We completely agree with these authors that the measured outcomes, H_2_ and CH_4_, not only reflect the type of carbohydrate consumed, but are the end result of a very complex fermentation stoichiometry that depends on the host’s capacity to digest and absorb nutrients, the dominance and metabolic activity of microbial species, and their interactions. However, the conclusion that fermentation gases are extremely limited parameters to study carbohydrate fermentation is largely based on human data, where eating pattern, environment, genetic variation, and the gut microbe interact and ultimately determine an individual’s response to the diet^58,59^. When these and other factors can be better controlled, as it is the case with animal models, the analysis of carbohydrate fermentation through H_2_ and CH_4_ quantification has much to offer. The fact that *in vitro* models to measure H_2_ and CH_4_ evolution are still developing and proposed as a tool to unravel the mechanisms behind the association between microbiota and host health^60^ is encouraging.

Overall, the applications of gas analysis within an indirect calorimetry system go beyond the arena of carbohydrate quality and nutritional studies, and may be used as a diagnosis tool in clinical practice^19,61,62^. It opens up new avenues not only in preclinical research in rodents, but also has potential in human-diet-microbiota interaction studies if such sensor technology is incorporated into indirect calorimetry chambers or ventilated hood systems.

## Conclusions

Using our customized indirect calorimetry system we were able to continuously quantify H_2_ production in mice as a reflection of the starch digestibility of the diet. H_2_ monitoring also allowed us to catch the earliest stages in the adaptation to carbohydrates of different digestibility, revealing a nuanced process with high inter-individual variation. Importantly, *in vivo* H_2_ production was significantly correlated with specific microbial taxa, including *Bacteroides* and *Parasutterella*. The implemented H_2_ and CH_4_ sensor-technology described here opens yet unmet avenues to study the effects of nutrition on microbiota in real time, not only in rodents, but potentially also in humans.

## Methods

### Coupling of hydrogen (H_2_) and methane (CH_4_) sensors into indirect calorimetry system

A PhenoMaster indirect calorimetry system (TSE Systems, Bad Homburg, Germany) was extended by coupling a Sensepoint XCD H_2_ gas analyser (Honeywell Analytics, Hegnau, Switzerland) and a CH_4_ gas analyser (ABB Automation GmbH, Frankfurt am Main, Germany) in a closed circuit in series in front of a Siemens High-Speed Sensor Unit containing the O_2_ and CO_2_ sensors. This order was chosen to prevent dilution of the sample with reference air, which is required by the Siemens unit. The H_2_ sensor has a stability of <±2% full scale deflection (fsd)/yr representing <2 ppm/yr as it was adjusted to a measuring range from 0 to 100 ppm. The CH_4_ sensor has a zero drift of ≤1% of span per week and a measuring range from 0 to 500 ppm. A two point calibration of both H_2_ and CH_4_ analysers was performed within 24 h before each animal experiment. The calibration procedure was carried out using three gas mixtures (Linde Gas Benelux BV, Dieren, The Netherlands): zero (20.947% O_2_ and N_2_), span H_2_ (98.8 ppm H_2_ and synthetic air), and span CH_4_ (0.521% CO_2_, 450 ppm CH_4_, and N_2_). The zero calibration mixture was flushed through the system for 10 min and ADC signals were assigned H_2_ and CH_4_ values of 0 ppm. Thereafter, each of the span gas mixtures was run for 10 min and ADC signals assigned 98.8 ppm H_2_ and 450 ppm CH_4_, accordingly. For O_2_ and CO_2_ calibration, the routine indicated in the TSE manufacturer’s manual was followed, using an additional gas mixture (0.999% CO_2_ and N_2_) for the span calibration point. Animals were measured as previously described^63^ with minor adjustments for the newly coupled sensors. These include the adjustment of airflow to 0.431 min^−1^ and the measuring time per cage set to 1.5 min. Data was recorded using an updated, customized, version of the TSE PhenoMaster software (V5.8.0) specially developed for the incorporation of H_2_ and CH_4_ measurements.

### Animal experiments and sample harvest

All animal experiments were approved by the Animal Experiments Committee (DEC 2014085.h) of Wageningen, The Netherlands, and performed in accordance to EU directive 2010/63/EU. Female and male C57BL/6JRccHsd mice (Harlan Laboratories BV, Horst, The Netherlands) were housed in Makrolon II cages enriched with wood chips and wood shavings, with free access to drinking water, at 23 °C ± 1 °C and a 12:12 h light:dark cycle. Standard rodent chow (RMH-B, AB Diets, Woerden, The Netherlands) was provided exclusively and continuously since weaning, unless specified. Three different studies were conducted to investigate diet-host-microbiota interactions upon provision of diets containing starches with differences in digestibility (the experimenter was not blinded to the diets that the animals were given).

#### Study 1 (long-term exposure, post-weaning)

Mice were mated and the offspring reassigned to a foster dam 1 or 2 days after birth to obtain standardized litters. Males and females were stratified by body weight at post-natal day (PN) 21, housed individually and randomly assigned to be fed a highly- or a lowly-digestible starch diet (HDD and LDD, respectively; see below). The randomisation was achieved by generating a column of random numbers in a spreadsheet and sorting each diet and animal number according to the column of random numbers from smallest to largest. From PN36-42, a subgroup of mice was measured in the indirect calorimetry system with *ad libitum* access to the experimental diets (males: n = 12 per diet, females HDD n = 12, LDD n = 11). Fresh faecal pellets were sampled on PN39 (n = 6 per diet and sex) and stored at −80 °C for intestinal microbiota analysis. Another subgroup of female mice was culled on PN42 for collection of caecum (n = 6) and colon contents (n = 5 HDD, n = 7 LDD), and the faeces produced during the last week before sacrifice were collected for gross energy measurements (see *In vivo* diet digestibility). Before sacrifice, food was removed 1 h after the start of the light phase and animals decapitated 2–6 h after removal of food. Caecum and colon contents were immediately frozen in liquid nitrogen, and stored at −80 °C until analysis.

#### Study 2 (short-term exposure with fasting, adult)

Eight-month-old female mice were individually housed in indirect calorimetry cages. After a 2-day adaptation period, mice were allowed a restricted amount (1.1 g) of chow 1 h before the onset of the dark phase to induce a fasting state in early morning, as published^64^. At the end of the light phase at 18.00 h, mice were re-fed with a restricted amount (1.1 g) of chow, or first-time HDD or LDD (the refeeding diet was assigned randomly; n = 4 per dietary group). Shortly before the following dark phase mice received access to the same diet they were allocated the day before, but now *ad libitum*. Indirect calorimetry measurements continued for an additional 5.5 d.

#### Study 3 (short-term exposure without fasting, adult)

Ten-month-old female mice were individually housed in indirect calorimetry cages. After a 2-day adaptation period, mice were provided clean bedding and given *ad libitum* first-time access to either HDD or LDD (random assignment, n = 6) shortly before the dark phase and for the remaining experimental period. Measurements continued for an additional 4.5 d. Faecal pellets produced after the introduction of the new diets were collected from the bedding at the end of the experiment and stored at −80 °C.

### Experimental diets

Both the HDD and the LDD satisfy the nutrient requirements for rodent growth and lactation (AIN-93G)^65^, with appropriate levels of mono- and poly-unsaturated fatty acids. The macronutrient composition was 20.1 energy percentage (en%) protein, 54.9 en% carbohydrates, and 25 en% fat (Table 1), with starch being the sole source of carbohydrates. The starch fraction (Cargill BV, Sas van Gent, The Netherlands) of the HDD was composed of 100% amylopectin (which is highly digestible), while that of the LDD was a mixture of 60% amylose (which resists complete digestion) and 40% amylopectin. The diets were pelleted by Research Diet Services BV, Wijk bij Duurstede, The Netherlands.

### *In vivo* diet digestibility

Total faeces produced from PN 35–42 (Study 1) were recovered from the bedding of a subgroup of randomly selected animals (*n* = 4 per sex and diet). Food intake was recorded over the same period. Gross energy in faeces and food was determined in blinded samples using a C7000 bomb calorimeter (IKA, Staufen, Germany) and diet digestibility was calculated as published^66^.

### *In vitro* carbohydrate digestibility

The *in vitro* digestibility of starches in the experimental diets was determined in blinded samples in triplicate, as published^67^. Briefly, 5 intact pellets of each diet were cryoground to homogeneous particle size and weighed separately into 3 tubes (70 mg). Each sample was digested in a 15-ml tube by adding cocktail solutions (modified from Versantvoort *et al*.^68^) and digestive enzymes at 37 °C in three sequential steps to represent the oral (5 min), gastric (2 h), and duodenal (6 h) phases. Two blanks containing only enzymes and solutions were included. Samples were taken at several time points during the gastric and duodenal phases and centrifuged. Clean supernatants were recovered and free glucose content was determined by the glucose oxidase peroxidase method^69^. Starch digestion was expressed as the percentage of total glucose released based on the amount of starches in the diets.

### Quantification of SCFA in intestinal digesta by gas chromatography (GC)

Short-chain fatty acids in caecum- and colon-contents were determined as previously reported^70^, with some modifications. Samples (about 25 mg) were weighed, thawed, homogenized in 100 μl of ultrapure water, and centrifuged for 3 min at 21,382 *g*. To 50 μl of supernatant, 100 μl of 2-ethylbutyric acid solution (0.45 mg ml^−1^) were added as internal standard. An external standard curve was prepared containing 50 μl of a mixture of acetic, propionic, butyric, valeric, isobutyric, and isovaleric acid at concentrations ranging from 0.002 mg ml^−1^ to 0.8 mg ml^−1^, to which 100 μl of internal standard were added. Blanks containing only water or water and internal standard were included for quality control. HCl and oxalic acid were added to all samples, blanks, and standards in order to protonate the SCFA. Gas chromatography was performed on a FOCUS GC apparatus coupled to a flame ionization detector (Interscience, Breda, The Netherlands). Samples were injected (1 μl) into an CP-FFAP CB column (25 m × 0.53 mm × 1.00 μm; Agilent Technologies, Santa Clara, CA, USA). Helium served as carrier gas at a pressure of 40 kPa. The initial oven temperature was 100 °C with 0.5 min hold, ramped to 180 °C at 8 °C min^−1^ with 1 min hold, and finally ramped to 200 °C at 20 °C min^−1^ with 5 min hold. Peak identities and areas were analysed with Xcalibur software (version 2.2; Thermo Scientific, Waltham, MA, USA). Concentrations of SCFA were normalised to the internal standard and expressed relative to original sample weight.

### Microbiota analysis

Microbial DNA was isolated from faecal pellets using the Maxwell® 16 Instrument (Promega, Leiden, The Netherlands). Faecal pellets were added to a bead-beating tube with 350 μl Stool Transport and Recovery (STAR) buffer, 0.25 g of sterilized zirconia beads (0.1 mm), and three glass beads (2.5 mm). Faecal pellets were homogenized by bead-beating three times (60 s × 5.5 ms) and incubation for 15 min at 95 °C at 100 rpm. Samples were then centrifuged for 5 min at 4 °C and 14,000 *g* and supernatants transferred to sterile tubes. Pellets were re-processed using 200 μl STAR buffer and both supernatants were pooled. DNA purification was performed with a customized kit (AS1220; Promega) using 250 μl of the final supernatant pool. DNA was eluted in 50 μl of DNAse- RNAse-free water and its concentration measured using a DS-11 FX+ Spectrophotometer/Fluorometer (DeNovix Inc., Wilmington, USA). The V4 region of 16S ribosomal RNA (rRNA) gene was amplified in duplicate PCR reactions for each sample in a total reaction volume of 50 μl. The master mix contained 1 μl of a unique barcoded primer, 515F-n and 806R-n (10 μM each per reaction), 1 μl dNTPs mixture, 0.5 μl Phusion Green Hot Start II High-Fidelity DNA Polymerase (2 U/μl; Thermo Scientific, Landsmeer, The Netherlands), 10 μl 5× Phusion Green HF Buffer, and 36.5 μl DNAse- RNAse-free water. The amplification program included 30 s of initial denaturation step at 98°C, followed by 25 cycles of denaturation at 98 ^o^C for 10 s, annealing at 50 °C for 10 s, elongation at 72 °C for 10 s, and a final extension step at 72 °C for 7 min. The PCR product was visualised in 1% agarose gel (~290 bp) and purified with CleanPCR kit (CleanNA, Alphen aan den Rijn, The Netherlands). The concentration of the purified PCR product was measured with Qubit dsDNA BR Assay Kit (Invitrogen, California, USA) and 200 ng of microbial DNA from each sample were pooled for the creation of the final amplicon library which was sequenced (150 bp, paired-end) on the Illumina HiSeq. 2000 platform (GATC Biotech, Constance, Germany).

### Microbiota data processing and analysis

Data filtering and taxonomy assignment were performed using the NG-Tax pipeline^71^. Briefly, an OTU table was created for each sample with the most abundant sequences. Low abundance OTUs were discarded, using a minimum relative abundance threshold of 0.1%. Two distinct in-house assembled mock communities were included in the library and were compared with their theoretical composition for quality control (Additional file 6: Fig. 6). Calculations for α- and β-diversity analyses were performed using the publicly available Microbiome R package (version 1.2.1)^72^. Adonis permutational multivariate analyses of variance (PERMANOVA) using either the weighted or unweighted Unifrac distances were performed with the Vegan package (version 2.5-2) and were used to determine the amount of variation explained by the grouping variables. Linear Discriminant Analysis (LDA) Effect Size (LEfSe) was applied to determine the differences between the microbial communities of HDD- and LDD-fed mice using a publicly available pipeline (http://huttenhower.sph.harvard.edu/galaxy/)^73^; the threshold for the logarithmic LDA score was set to 2.0. *P* values for Kruskal-Wallis and Wilcoxon tests for the LEfSe analysis were set to 0.05. For non-parametric Student’s *t*-tests, reads were transformed to their relative abundances and tests were carried out with 999 permutations using QIIME (version 1; http://qiime.org/index.html)^74^. Statistical significance was determined using the Benjamini-Hochberg false discovery rate (FDR) adjustment.

### Data analysis

Statistical analysis was performed using GraphPad Prism 5.04 (GraphPad, San Diego, CA, USA), unless stated otherwise. All data was tested for normality using the D’Agostino and Pearson omnibus test and its distribution was normalized by log transformation when applicable. Comparisons between two groups were made using unpaired two-tailed Student’s *t*-tests (for data with normal distribution) or two-tailed Mann-Whitney *U* tests (VH_2_ during light phase between HDD and LDD). Comparisons between more than two groups were made by one-way analysis of variance (ANOVA) with *post hoc* Bonferroni’s test for multiple comparisons. Time course data (H_2_ evolution) was analysed by repeated measures two-way ANOVA with Bonferroni’s *post hoc* test. When sample sizes being compared were similar and relatively large (n > 5), similarity of variances was not taken into account. All data is reported as mean ± s.d. Statistical significance was set at 5%, with levels indicated as *P ≤ 0.05, **P ≤ 0.01, ***P ≤ 0.001, and ****P < 0.0001. Sample size was not determined statistically as the effect size was unknown, but it was based on our previous results on the use of indirect calorimetry to assess metabolic flexibility^64,75^.

### Ethics approval and consent to participate

All animal experiments were approved by the Animal Experiments Committee (DEC 2014085.h) and performed in accordance to EU directive 2010/63/EU.

## Electronic supplementary material

Supplementary information

## Data Availability

The 16S rRNA gene sequencing dataset supporting the conclusions of this article is available in the European Nucleotide Archive (ENA) database with accession code PRJEB23475 at http://www.ebi.ac.uk/ena/data/view/prjeb23475. The authors declare that all other data supporting the findings of this study are available within the paper and its additional files 1–6, or from the corresponding authors upon reasonable request.
