# Supplementary material for: Non-invasive continuous real-time in vivo analysis of microbial hydrogen production shows adaptation to fermentable carbohydrates in mice
Source: Sci Rep. 2018 Oct 18;8:15351. doi: 10.1038/s41598-018-33619-0 (PMC6193968; doi:10.1038/s41598-018-33619-0)
Supplement: Supplementary file 1 — Supplementary information [file 41598_2018_33619_MOESM1_ESM.pdf]

Supplementary information for:

Non-invasive continuous real-time *in vivo* analysis of microbial hydrogen production shows adaptation to fermentable carbohydrates in mice

José M. S. Fernández-Calleja[1]; Prokopis Konstanti[2]; Hans J. M. Swarts[1]; Lianne M. S. Bouwman[1]; Vicenta Garcia-Campayo[3]; Nils Billecke[4]; Annemarie Oosting[5]; Hauke Smidt[2]; Jaap Keijer[1]; & Evert M. van Schothorst[1]*

[1]Human and Animal Physiology, Wageningen University, De Elst 1, Wageningen 6708 WD, The Netherlands

[2]Laboratory of Microbiology, Wageningen University, Stippeneng 4, Wageningen 6708 WE, The Netherlands

[3]Cargill R&D, Minneapolis, MN, USA

[4]Cargill R&D Centre Europe, Havenstraat 84, Vilvoorde 1600, Belgium

[5]Danone Nutricia Research, Uppsalalaan 12, Utrecht 3584 CT, The Netherlands

Supplementary Figures S1 - S6

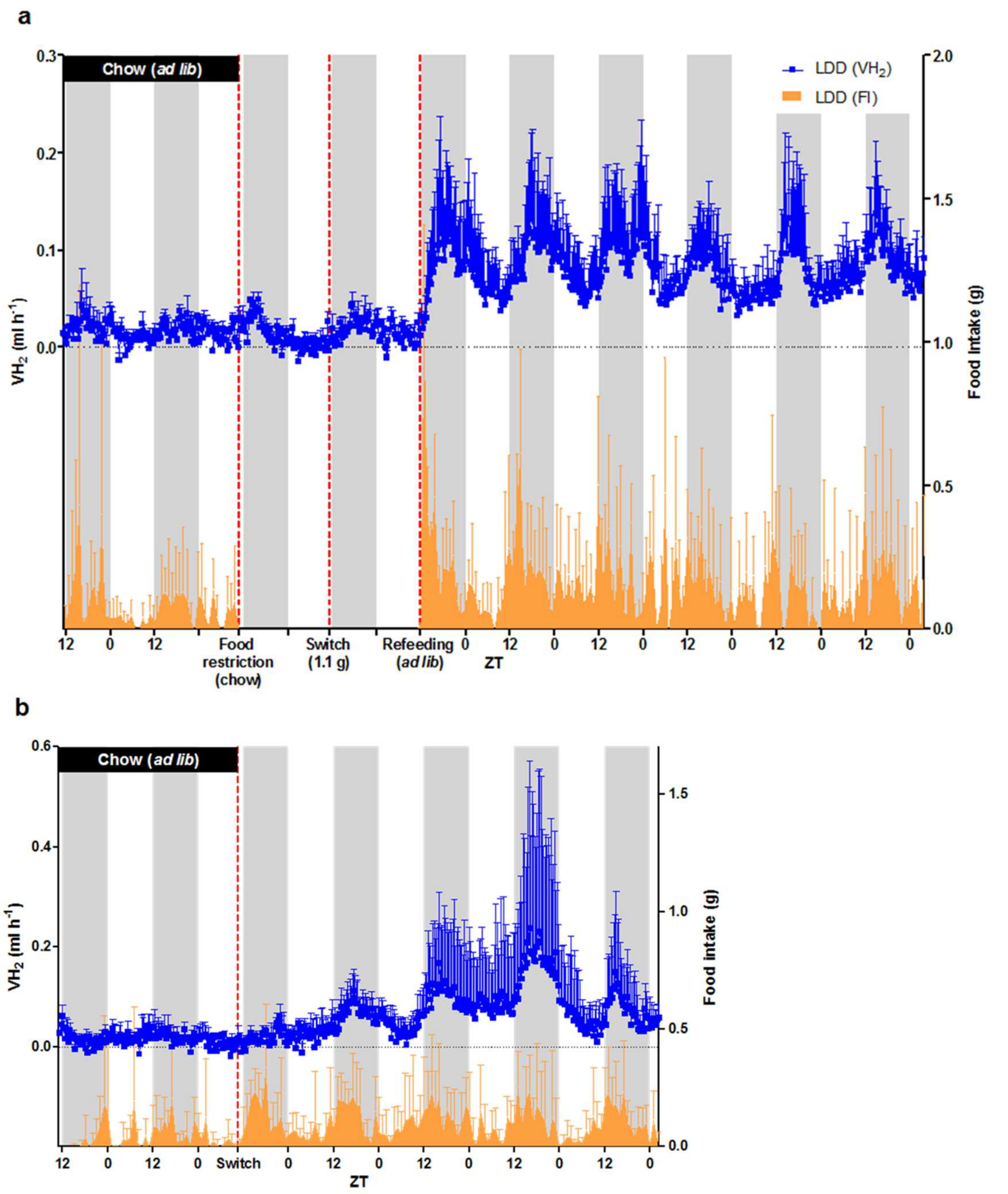

Supplementary Figure S1. Hydrogen (H_2) production closely follows food intake (FI) patterns. (a) Mice within indirect calorimetry were fed standard chow, followed by chow restriction leading to a fasting state (left dotted line), which was subsequently followed by feeding 1.1 g of LDD (blue; *n*=4) prior to the dark phase as a single meal test (2nd dotted line). As a result, mice were fasted the next day, and received prior to next dark phase *ad libitum* access to LDD (3rd dotted line) for an additional 5.5 d. (b) Chow-fed mice (*n*=6) were switched to LDD without prior food restriction and measurements continued for another 4.5 d. All mice received no other diet than chow during their whole lifetime prior to these experiments until the dietary switch (black bar), but colour usage reflects exposure to new diets. Volume of H_2 produced (VH_2; blue squares, left y-axis, ml h^{-1}) is plotted together with associated hourly food intake episodes (orange area, right y-axis, g), except when food was placed directly inside the cage and not in the food baskets (food restriction, panel a). White and grey areas represent light and dark phases, respectively. Data is presented as mean ± s.d. For clarity, only upper error bars are shown. ZT, Zeitgeber time.

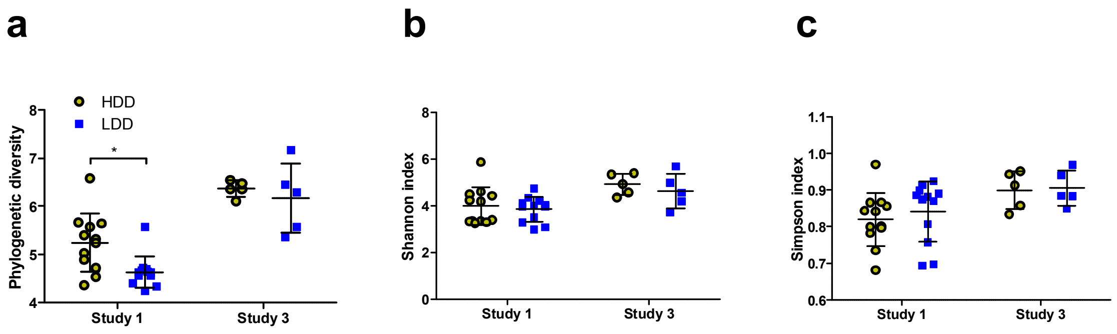

Supplementary Figure S2. Metrics of α-diversity in faecal microbiota of HDD- or LDD-fed mice. Phylogenetic diversity (a), and Shannon (b) and Simpson (c) indexes of mice fed HDD or LDD for 3 weeks (*n*=12 per diet, Study 1) or 4.5 d (*n*=5 per diet, Study 3). Statistical comparisons were made using unpaired two-tailed Student's *t*-test; *$P \leq 0.05$. Data shown as mean ± s.d.

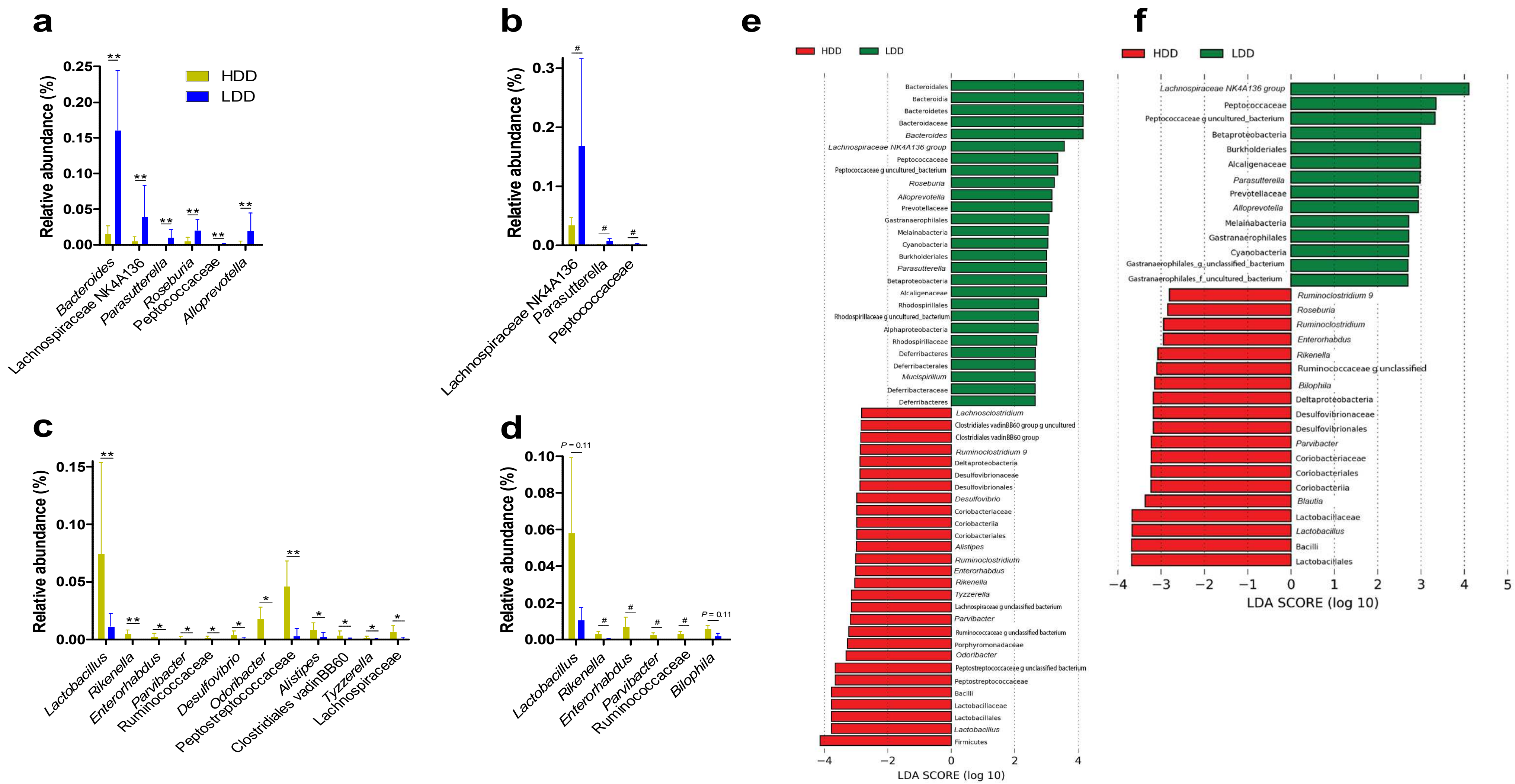

Supplementary Figure S3. Exposure to starches of different digestibility induces distinct microbial taxa. All bacterial taxa that were significantly increased by LDD (a) or HDD (c) after 3 weeks of exposure to the diets (*n*=12 per diet, Study 1) are shown. All taxa that were significantly increased by LDD (b) or HDD (d) after 4.5 d of exposure to the diets (*n*=5 per diet, Study 3) are also shown for comparison. Side-by-side boxes represent the $\log_{10}$ transformed LDA scores of bacterial taxa enriched in HDD- or LDD-fed mice in Study 1 (e) and Study 3 (f) analysed by LEfSe. Genera are indicated in italics, uncultured genera are indicated by family name in regular font. Comparisons were done by non-parametric *t*-test followed by 999 permutations and *P* values were adjusted by False Discovery Rate (FDR) correction; $^{\#}P < 0.1$, $^{*}P \leq 0.05$, $^{**}P \leq 0.01$. Data is presented as mean ± s.d.

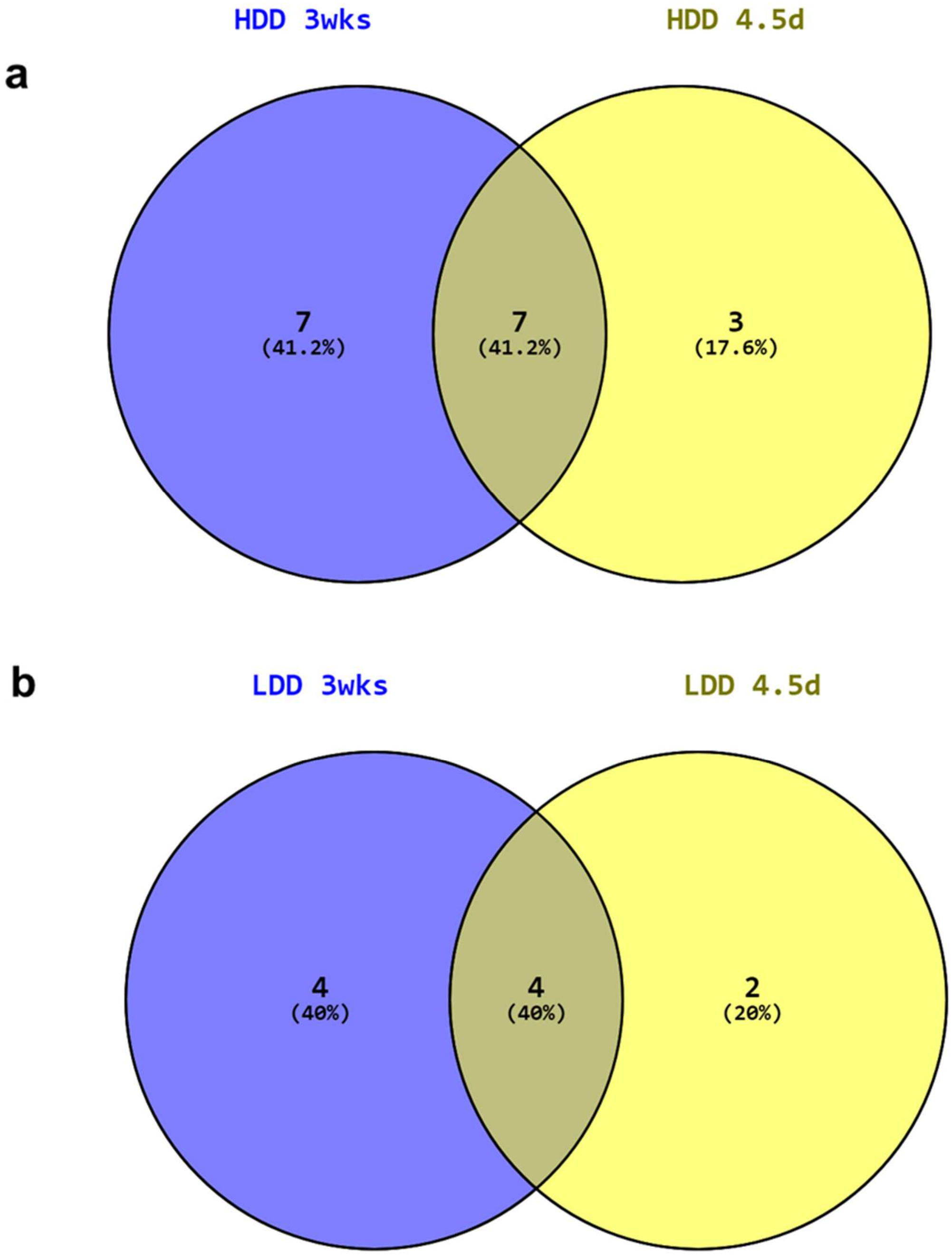

Supplementary Figure S4. Venn diagrams showing the unique and shared genera which are significantly enriched as determined by the LefSe analysis between mice a) exposed to HDD and b) to LDD.

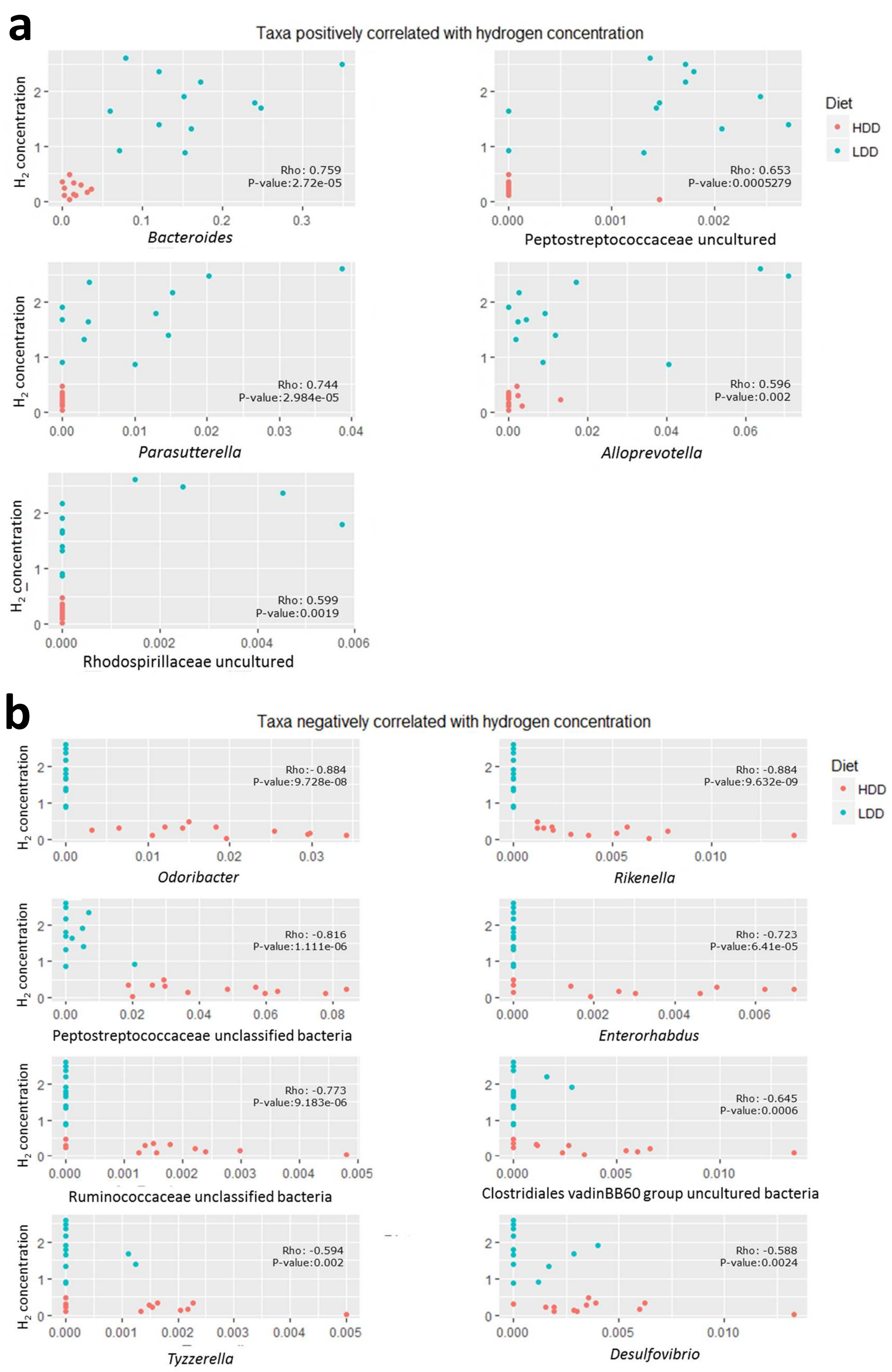

Supplementary Figure S5. Specific bacterial genera correlating with *in vivo* H_2 production. (a) Positive and (b) negative Spearman's rank correlations of relative abundance (%) of specific faecal bacterial genera (x-axis) and 24 hr H_2 production (ml; y-axis) of mice exposed to HDD or LDD for 3 weeks after weaning (*n*=12 per diet, Study 1); FDR threshold set to *P* < 0.1.

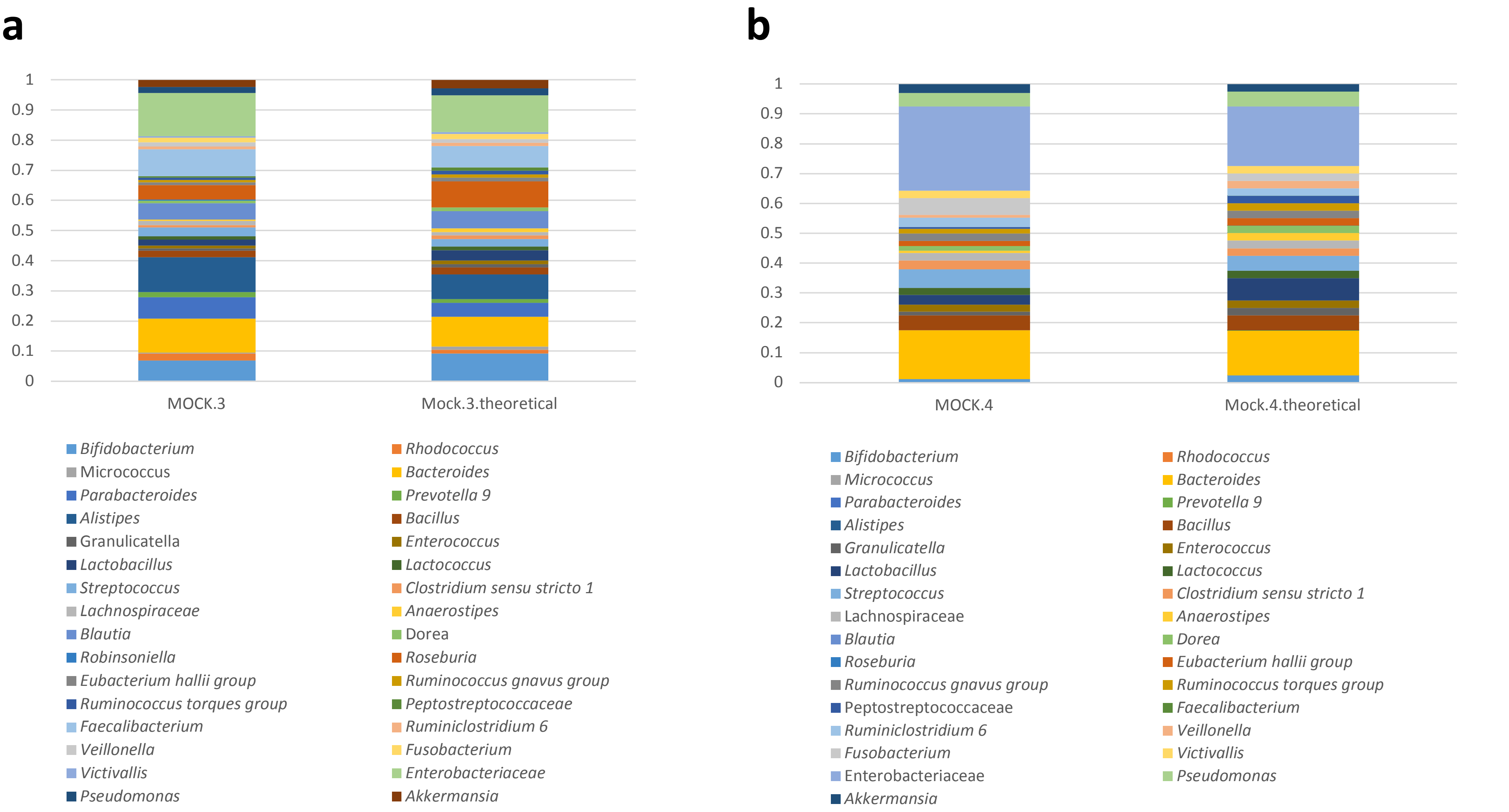

Supplementary Figure S6. Bacterial composition of the two synthetic mock communities which were used as controls for sequencing run (“mock 3”, a; “mock 4”, b). Both mock communities were compared against their theoretical composition using non-parametric Pearson correlation showing high similarity. Correlation coefficients are 0.935 for mock 3 and 0.96 for mock 4, respectively.
